# Protective Effects of Arecoline on LPS-Induced Neuroinflammation in BV2 Microglial Cells

**DOI:** 10.3390/ijms262412097

**Published:** 2025-12-16

**Authors:** Xiangfei Zhang, Jingwen Cui, Jing Sun, Bei Fan, Fengzhong Wang, Cong Lu

**Affiliations:** 1 Institute of Food Science and Technology, Chinese Academy of Agricultural Sciences, Beijing 100193, China; 2 Institute of Food and Nutrition Development, Ministry of Agriculture and Rural Affairs, Beijing 100081, China

**Keywords:** arecoline, *Areca catechu*, bioactive alkaloid, BV2 microglia, neuroinflammation, microglial activation, signaling pathways

## Abstract

Natural alkaloids derived from edible and medicinal plants have recently gained attention as bioactive molecules capable of modulating neuroinflammatory processes. Arecoline, the major alkaloid constituent of *Areca catechu* L. (betel nut), is well known for its cholinergic actions, yet its direct regulatory influence on microglial immune signaling has remained uncertain. In this study, murine BV2 microglial cells were employed to investigate whether arecoline could counteract lipopolysaccharide (LPS)-induced neuroinflammatory responses. Parameters including cell viability, nitric oxide (NO) production, cytokine secretion, and gene expression were assessed, and mechanistic analyses were focused on the Toll-like receptor 4 (TLR4)/nuclear factor-κB (NF-κB) and phosphoinositide 3-kinase (PI3K)/protein kinase B (AKT) pathways. Non-toxic doses of arecoline (10–40 μmol/L) markedly decreased NO accumulation and reduced the expression of tumor necrosis factor-α (TNF-α), interleukin-6 (IL-6), and interleukin-1β (IL-1β). Western blot analysis further showed that arecoline suppressed LPS-activated microglial signaling by down-regulating TLR4, inhibiting NF-κB p65 phosphorylation, and limiting PI3K/AKT activation. Collectively, these data reveal that arecoline exerts immunomodulatory and neuroprotective effects through dual signaling regulation in microglia and may serve as a useful pharmacological tool or structural reference for elucidating microglial inflammatory regulation and for guiding the exploration of safer bioactive compounds.

## 1. Introduction

Neuroinflammation has been increasingly acknowledged as a key driver in the onset and advancement of neurodegenerative disorders, including Alzheimer’s disease, Parkinson’s disease, and major depressive disorder [1,2]. The excessive generation of inflammatory mediators—including tumor necrosis factor-α (TNF-α), interleukin-6 (IL-6), interleukin-1β (IL-1β), and nitric oxide (NO)—disrupts neuronal stability and synaptic function, ultimately causing irreversible injury to the central nervous system (CNS) [3,4,5]. Because microglia are the primary cellular source of these mediators, targeting microglial activation is considered a central therapeutic strategy in neuroinflammation-related diseases [6,7].

Microglia act as the intrinsic immune sentinels of the CNS, continuously surveilling the neural environment under normal conditions [8,9]. Upon pathogenic stimulation or injury, they rapidly adopt an activated phenotype characterized by elevated release of cytokines and other inflammatory effectors [10,11]. Chronic microglial activation contributes to sustained inflammatory signaling and neuronal dysfunction [12,13]. Among experimental models, the murine BV2 microglial line stimulated by lipopolysaccharide (LPS) provides a reproducible system for mimicking inflammatory activation in vitro and is widely used for screening potential anti-inflammatory agents [14,15]. This multilayered signaling cascade underscores the need for modulators capable of targeting multiple nodes within the microglial inflammatory network. Because NO production downstream of iNOS is a major effector and a readily quantifiable marker of microglial activation, LPS-induced NO release is commonly used as a functional readout of inflammatory magnitude in BV2 cells [16,17,18].

Natural products and their bioactive constituents have emerged as rich sources of neuroprotective and immunomodulatory compounds [19,20]. Owing to their structural diversity and multitarget properties, many plant-derived molecules are being explored as bioactive compounds for managing oxidative and inflammatory damage in the CNS [21]. Alkaloids, in particular, represent an important class of bioactive molecules with potent antioxidant, anti-inflammatory, and neuroprotective activities [22]. Arecoline, the predominant alkaloid from *Areca catechu* (betel nut) [23], has been reported to modulate learning, memory, and mood-related behaviors in animal models [24,25,26]. Its pharmacological profile indicates that arecoline can influence cholinergic signaling and CNS-related behaviors. Notably, despite these reported CNS activities, arecoline also possesses well-documented toxicological properties, which limit its direct translational application. Nevertheless, its unique bioactivity profile makes it a valuable molecular scaffold for mechanistic studies of neuroimmune regulation.

A growing body of evidence indicates that neuroinflammation is closely intertwined with cognitive and emotional dysfunction [27,28], raising the possibility that the CNS effects of arecoline may involve modulation of microglial inflammatory signaling. Because arecoline is lipid-soluble and capable of blood–brain barrier penetration [29,30], it may directly interact with CNS innate immune cells. However, current studies on arecoline primarily focus on neurotransmission and behavioral regulation, whereas its direct influence on microglial immune pathways has not been systematically investigated. This represents a key knowledge gap, particularly given the central role of microglial activation in neuroinflammatory disorders.

Therefore, this study investigated whether arecoline modulates inflammatory activation in LPS-stimulated BV2 microglia, with a focus on the TLR4/NF-κB and PI3K/AKT signaling pathways. By defining its cellular and molecular effects, we aim to clarify how this alkaloid influences microglial immune responses and provide mechanistic insight into its neuroimmune regulatory potential.

## 2. Results

### 2.1. Effects of LPS on Microglial Viability and NO Production, and Cytotoxicity Assessment of Arecoline

**Figure 1:**
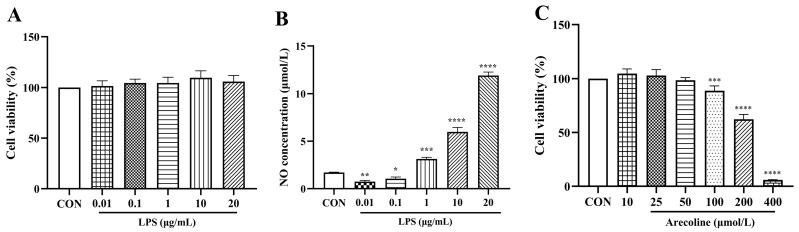
Effects of LPS on microglial viability and NO production, and cytotoxicity assessment of arecoline. (**A**) Cell viability after 24 h exposure to different concentrations of LPS (0.01–20 μg/mL). (**B**) Concentration-dependent increase in NO release induced by LPS. (**C**) Cell viability after 24 h treatment with arecoline (10–400 μmol/L). Values represent mean ± SEM (*n* = 5). The CON group, set at 100% for normalization in the cell viability assay, displays no error bars. Statistical analysis was performed using one-way ANOVA followed by Tukey’s multiple-comparison test. * *p* < 0.05, ** *p* < 0.01, *** *p* < 0.001, **** *p* < 0.0001 vs. control.

As illustrated in Figure 1A, LPS exposure did not significantly influence cellular viability across this range (*p* > 0.05), suggesting that BV2 cells tolerate LPS without measurable cytotoxicity. However, LPS markedly stimulated NO synthesis in a dose-dependent manner (Figure 1B). Even the lowest concentrations (0.01–0.1 μg/mL) produced a significant rise in NO relative to untreated controls (*p* < 0.05–0.01), and further increases were observed at 1–20 μg/mL (*p* < 0.001–0.0001). This slight reduction in NO at 0.01–0.1 μg/mL LPS is consistent with previous reports demonstrating that low-dose LPS preconditioning (or priming) can induce an immune-tolerant or hypo-responsive state in microglia, leading to suppression—rather than activation—of iNOS/NO synthesis [31]. Based on these findings, 1 μg/mL LPS was selected for subsequent experiments because it elicited a robust inflammatory response while maintaining cellular integrity.

To determine appropriate non-cytotoxic concentrations of arecoline for subsequent assays, we further evaluated its effects on BV2 viability. As shown in Figure 1C, arecoline did not significantly alter cell viability at 10–25 μmol/L, and only a slight, non-significant reduction was observed at 50 μmol/L. In contrast, higher concentrations (≥100 μmol/L) produced a clear, concentration-dependent decline in viability, with marked cytotoxicity at 200 μmol/L and near-complete loss of viability at 400 μmol/L. Based on these results, 10, 20, and 40 μmol/L were selected as safe working concentrations for the experiments described in Section 2.2.

### 2.2. Arecoline Maintains BV2 Microglial Viability and Reduces LPS-Induced Cytotoxicity

**Figure 2:**
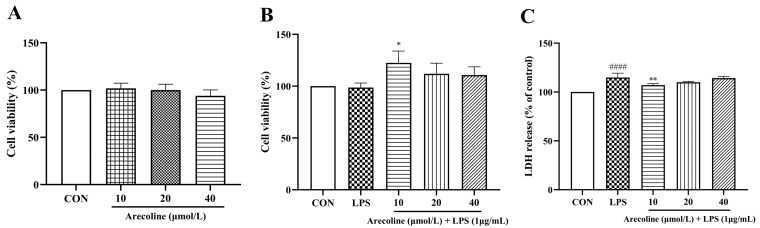
Effects of arecoline on BV2 microglial viability and membrane integrity with or without LPS stimulation. (**A**) Cell viability after 24 h incubation with arecoline (10–40 µmol/L). (**B**) Cell viability following combined treatment with arecoline (10–40 µmol/L) and LPS (1 µg/mL) for 24 h. (**C**) LDH release in BV2 cells treated with LPS (1 µg/mL) alone or in combination with arecoline (10–40 µmol/L) for 24 h, reflecting plasma membrane integrity. Values represent mean ± SEM (*n* = 5). The CON group, set at 100% for normalization in the cell viability assay, displays no error bars. Statistical analysis was performed using one-way ANOVA followed by Tukey’s multiple-comparison test. #### *p* < 0.0001 vs. control; * *p* < 0.05, ** *p* < 0.01 vs. control.

Consistent with the cytotoxicity assessment, arecoline showed no cytotoxic effects within the concentration range used in this study (10–40 μmol/L), indicating that the applied doses are non-cytotoxic to BV2 microglia. Figure 2A indicates arecoline (10–40 μmol/L) had no impact on BV2 cell viability versus controls (*p* > 0.05). These results confirm that arecoline alone does not alter the basal viability or activation state of BV2 microglia under non-inflammatory conditions. When cells were co-treated with LPS (1 μg/mL), low-dose arecoline (10 μmol/L) slightly increased viability relative to the LPS group (*p* < 0.05), suggesting mild cytoprotection under inflammatory stress (Figure 2B). However, higher concentrations (20 and 40 μmol/L) failed to produce further improvement, indicating that the viability-enhancing effect of arecoline occurred within a limited concentration range. In line with the CCK-8 assay, LDH release showed that LPS (1 µg/mL) induced a modest increase in membrane damage, whereas arecoline (10–40 µmol/L) did not further enhance LDH release compared with the LPS group, indicating no overt cytotoxicity at the concentrations used (Figure 2C). Overall, arecoline maintained BV2 cell viability, showing no cytotoxic effects with or without LPS stimulation.

### 2.3. Arecoline Attenuates LPS-Stimulated Nitric Oxide Production in BV2 Microglia

**Figure 3:**
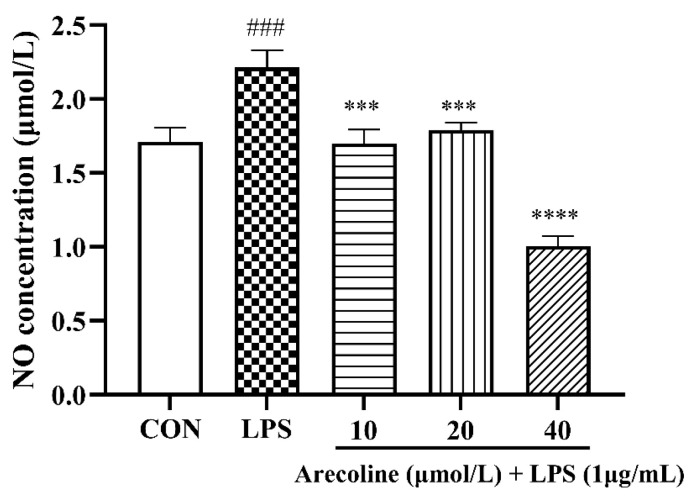
Quantification of nitric-oxide generation in BV2 microglia pre-treated with arecoline. Values represent mean ± SEM (*n* = 5). Statistical analysis was performed using one-way ANOVA followed by Tukey’s multiple-comparison test. ### *p* < 0.001 vs. control; *** *p* < 0.001, **** *p* < 0.0001 vs. LPS group.

LPS (1 μg/mL) treatment of BV2 microglial cells for 24 h significantly elevated NO levels versus the control (*p* < 0.001) (Figure 3). Arecoline co-treatment effectively attenuated LPS-stimulated NO production, and this inhibitory effect became prominent at the highest concentration tested (40 μmol/L). At 10 μmol/L and 20 μmol/L, NO levels were moderately reduced (*p* < 0.001 vs. LPS), whereas 40 μmol/L arecoline produced a pronounced inhibition (*p* < 0.0001). These results demonstrate arecoline effectively attenuates excessive NO generation triggered by LPS stimulation in BV2 microglia.

### 2.4. Effect of Arecoline on LPS-Induced Inflammatory Cytokine Release in BV2 Cells

**Figure 4:**
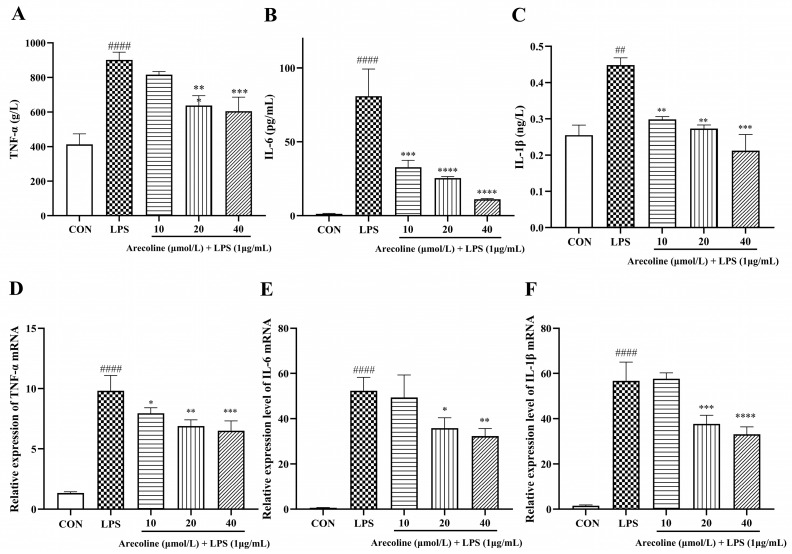
Assessment of inflammatory mediators in BV2 microglia. (**A**–**C**) Protein concentrations of TNF-α, IL-6, and IL-1β in supernatants determined by ELISA. (**D**–**F**) Corresponding mRNA expression analyzed by qPCR, relative expression calculated by 2^−ΔΔCt^ and normalized to β-actin. BV2 cells received arecoline pretreatment (10–40 µmol/L) for two hours prior to 24-h LPS stimulation (1 µg/mL). Values represent mean ± SEM (*n* = 5). Statistical analysis was performed using one-way ANOVA followed by Tukey’s multiple-comparison test. ## *p* < 0.01, #### *p* < 0.0001 vs. control; * *p* < 0.05, ** *p* < 0.01, *** *p* < 0.001, **** *p* < 0.0001 vs. LPS group.

As depicted in Figure 4A–C, LPS substantially ramped up the secretion of inflammatory mediators TNF-α, IL-6, and IL-1β relative to untreated controls (*p* < 0.001). Co-treatment with arecoline significantly reduced cytokine levels in a concentration-dependent manner. At 10 μmol/L, arecoline moderately decreased TNF-α and IL-6 secretion (*p* < 0.05–0.01), whereas 20 μmol/L and 40 μmol/L resulted in further suppression of all three cytokines (*p* < 0.001–0.0001).

The mRNA expression pattern of TNF-α, IL-6, and IL-1β (Figure 4D–F) generally mirrored the ELISA results, showing significant LPS-induced up-regulation (*p* < 0.001) and arecoline-mediated suppression. Although the 10 μmol/L group exhibited a slight deviation, cytokine expression was consistently reduced at 20 μmol/L and 40 μmol/L (*p* < 0.001–0.0001). These results collectively suggest that arecoline mitigates LPS-induced inflammatory activation in BV2 cells at both gene expression and protein production.

### 2.5. Effect of Arecoline on LPS-Induced TLR4/NF-κB Signaling Pathway Proteins in BV2 Cells

**Figure 5:**
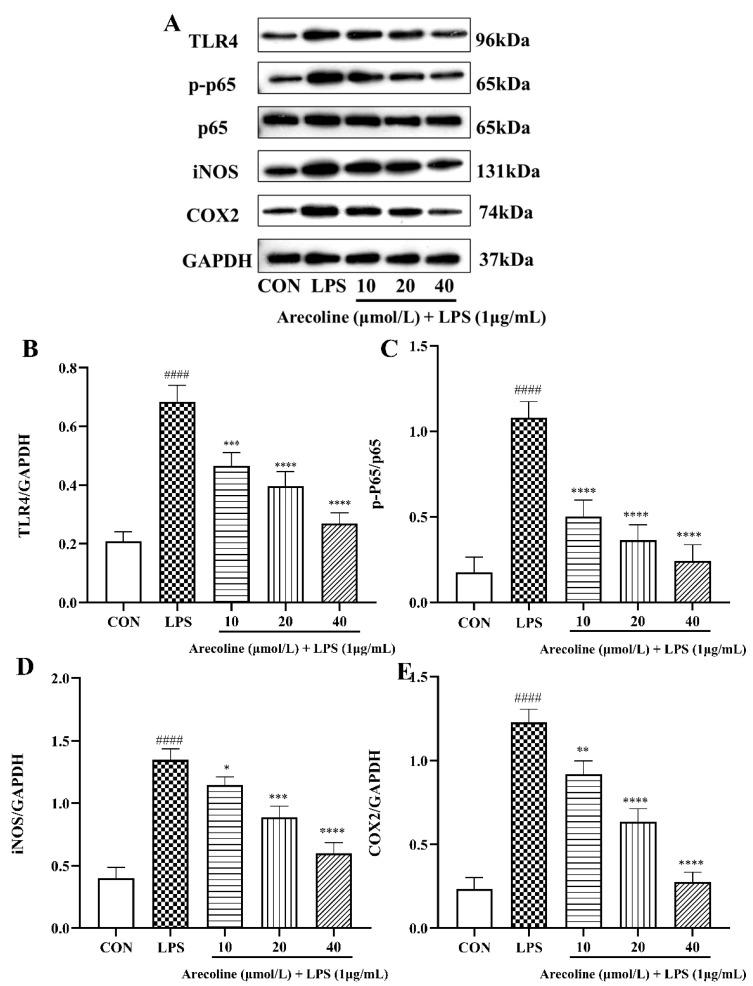
Expression of TLR4/NF-κB-related proteins in BV2 microglia. (**A**) Western blot bands. (**B**–**E**) Quantification of TLR4/GAPDH, p-p65/p65, iNOS/GAPDH, and COX2/GAPDH ratios. Values represent mean ± SEM (*n* = 3). Statistical analysis was performed using one-way ANOVA followed by Tukey’s multiple-comparison test. #### *p* < 0.0001 vs. control; * *p* < 0.05, ** *p* < 0.01, *** *p* < 0.001, **** *p* < 0.0001 vs. LPS group.

Western blot analysis revealed that LPS markedly up-regulated the protein expression of TLR4, phosphorylated NF-κB p65 (p-p65), iNOS, and COX-2 compared with the control group (*p* < 0.0001) (Figure 5A–E). Co-treatment with arecoline effectively attenuated these elevations in a dose-dependent fashion. Illustrated in Figure 5B,C, arecoline (10–40 μmol/L) significantly reduced TLR4 and p-p65 expression relative to the LPS group (*p* < 0.001–0.0001), indicating inhibition of upstream TLR4/NF-κB activation. Similarly, the levels of iNOS and COX-2 were also markedly suppressed by arecoline in a dose-dependent pattern (Figure 5D,E). At 20 μmol/L and 40 μmol/L, both iNOS and COX-2 expression decreased to near-baseline levels (*p* < 0.001–0.0001). These results demonstrate that arecoline down-regulates the TLR4/NF-κB signaling pathway and its associated downstream inflammatory elements within LPS-activated BV2 microglial cells, consequently diminishing the generation of pro-inflammatory enzymes and mitigating neuroinflammatory responses under laboratory conditions.

### 2.6. Effect of Arecoline on LPS-Induced PI3K/AKT Signaling Pathway Proteins in BV2 Cells

**Figure 6:**
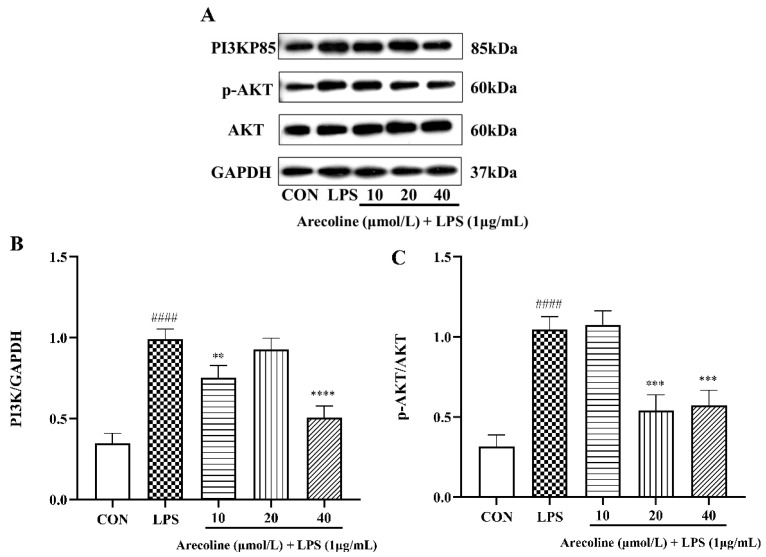
Expression of PI3K/AKT-related proteins in BV2 microglia. (**A**) Western blot bands. (**B**,**C**) Quantification of PI3K/GAPDH and p-AKT/AKT ratio. Values represent mean ± SEM (*n* = 3). Statistical analysis was performed using one-way ANOVA followed by Tukey’s multiple-comparison test. #### *p* < 0.0001 vs. control; ** *p* < 0.01, *** *p* < 0.001, **** *p* < 0.0001 vs. LPS group.

Figure 6A–C illustrate that LPS-induced treatment notably increased PI3K p85 and p-AKT expression versus the control (*p* < 0.001), with arecoline co-administration diminishing the response. At 10 μmol/L, arecoline slightly reduced PI3K p85 expression (*p* < 0.01), whereas 20 μmol/L and 40 μmol/L treatments caused further decreases, with the highest dose almost restoring protein levels to baseline (*p* < 0.0001 vs. LPS). The p-AKT/AKT ratio demonstrated a comparable response, showing significant suppression at both 20 μmol/L and 40 μmol/L doses of arecoline. The findings reveal that arecoline effectively inhibits LPS-induced PI3K/AKT signaling activation in BV2 microglial cells, suggesting that suppression of this pathway may contribute to its anti-inflammatory activity in vitro.

## 3. Discussion

Neuroinflammation has emerged as a defining characteristic across both neurodegenerative and neuropsychiatric conditions, where the overactivation of microglia combined with the unchecked release of inflammatory molecules contributes to neuronal dysfunction [32,33]. Accordingly, strategies aimed at limiting microglial hyperactivation have been widely regarded as a key therapeutic strategy to prevent or slow neurodegenerative progression [34]. In this context, we employed an LPS-induced BV2 microglial model to evaluate the effects of arecoline, the major alkaloid of *Areca catechu*. Our results showed that arecoline maintained microglial viability under inflammatory stress, reduced nitric-oxide and pro-inflammatory cytokine production, and inhibited two convergent signaling cascades—TLR4/NF-κB and PI3K/AKT—that are central to microglial activation. This dual-pathway suppression indicates that arecoline exerts coordinated regulation across both receptor-level and intracellular kinase signaling nodes, extending its pharmacological relevance beyond classical cholinergic effects and implying potential value in the management of neuroinflammation-related conditions.

Nitric oxide (NO) functions as a pivotal effector in neuroinflammatory processes, being synthesized primarily by activated microglia via the iNOS pathway [35,36]. Overproduction of NO promotes oxidative stress [37], mitochondrial impairment [38], and neuronal injury [39], and is therefore considered a hallmark of pathological microglial activation [34]. Consistent with previous evidence, LPS stimulation markedly elevated NO release in BV2 cells without compromising viability [40,41]. Arecoline exposure attenuated LPS-stimulated NO accumulation, with a marked suppression observed at the highest concentration (40 μmol/L). This concentration-dependent reduction indicates that arecoline may fine-tune microglial inflammatory responses rather than broadly suppressing cellular activity, suggesting potential modulation of upstream iNOS-associated regulatory processes.

Because NO synthesis is tightly linked to cytokine signaling [42,43], we next examined whether arecoline modulates cytokine expression. Pro-inflammatory cytokines like TNF-α, IL-6, and IL-1β mediate communication between activated microglia and neurons [44,45]. Their overproduction disrupts neuronal homeostasis, leading to synaptic dysfunction and neurotoxicity [46,47]. As reported previously [48,49], LPS stimulation markedly increased both transcription and secretion of TNF-α, IL-6, and IL-1β in BV2 microglia. Arecoline administration at non-cytotoxic concentrations markedly decreased these cytokine levels in a dose-dependent manner, particularly at higher doses. Notably, cytokine protein levels did not fully parallel mRNA expression; at 10 μmol/L arecoline, IL-6 and IL-1β proteins were decreased despite slightly elevated transcripts, a pattern consistent with post-transcriptional regulation and threshold-type suppression during LPS activation [50,51]. The stronger inhibitory effects observed at 40 μmol/L arecoline align with the known ability of cholinergic modulators to suppress NF-κB/iNOS signaling in a dose-dependent manner [52]. Together, these findings indicate that arecoline regulates cytokine output through coordinated modulation of multiple upstream signaling nodes. To place these effects into a broader inflammatory framework, LPS activates microglia through a multilayered cascade involving TLR4–MyD88/TRIF signaling and downstream amplification via mitochondrial dysfunction, ROS generation, and inflammasome activation [53,54,55,56]. Although our study focused on TLR4/NF-κB and PI3K/AKT, the concurrent inhibition of these pathways by arecoline suggests that it may attenuate several interconnected components of the LPS-driven inflammatory response.

Beyond cytokine suppression, arecoline exerted dual-site modulation of inflammatory signaling. TLR4 activation represents a primary trigger for microglial inflammatory transcription, leading to IκB degradation, NF-κB translocation, and subsequent induction of pro-inflammatory genes [57,58]. Consistent with these canonical mechanisms, LPS markedly increased TLR4 expression and p65 phosphorylation in BV2 microglia [59,60]. Arecoline pretreatment significantly attenuated both, suggesting inhibitory actions at the receptor level as well as on downstream transcriptional activation. This coordinated suppression suggests that arecoline acts at multiple mechanistic checkpoints within the TLR4/NF-κB signaling axis, contributing substantially to its observed anti-inflammatory phenotype.

The PI3K/AKT pathway represents a second major regulatory axis governing microglial activation, controlling both cellular survival and cytokine output [61,62]. Consistent with previous findings [63,64], LPS robustly activated this pathway, as evidenced by increased PI3K expression and AKT phosphorylation. Arecoline reduced both markers in a concentration-dependent manner, indicating that PI3K/AKT inhibition also contributes to its anti-inflammatory profile. The concurrent attenuation of TLR4/NF-κB and PI3K/AKT signaling suggests a coordinated mechanism in which arecoline modulates both receptor-initiated and intracellular kinase pathways. This dual-pathway inhibition may be particularly advantageous given the extensive cross-talk between TLR4 and PI3K/AKT pathways in shaping microglial activation dynamics. It is worth noting that basal activity of TLR4, NF-κB, PI3K, and AKT is typically low in resting microglia and becomes prominent chiefly under inflammatory stimulation such as LPS, hypoxia, or injury [61,65]. Because we did not assess pathway activity in unstimulated cells, future work employing more sensitive quantitative methods will be required to determine whether arecoline exerts regulatory effects under non-inflammatory conditions.

Several plant-derived alkaloids have previously been shown to suppress LPS-induced activation of microglia, providing relevant pharmacological context for the present findings. Tetrandrine, for example, has been reported to inhibit LPS-induced microglial activation by decreasing nitric oxide and superoxide production and reducing TNF-α and IL-6 release in parallel with suppression of NF-κB activation [66]. Matrine and related matrine-type alkaloids have also been implicated in anti-neuroinflammatory actions, at least partly through modulation of TLR4/NF-κB-related pathways [67,68]. Evodiamine attenuates LPS-induced inflammation in BV2 cells by down-regulating iNOS and pro-inflammatory cytokines via the AKT/Nrf2-HO-1/NF-κB signaling axis [69]. Although these compounds share anti-inflammatory actions, most operate through more restricted signaling routes. In contrast, arecoline inhibited both TLR4/NF-κB and PI3K/AKT pathways while simultaneously reducing NO and cytokine output, indicating a broader multi-pathway regulatory profile. These distinctions underscore arecoline’s utility as a mechanistically informative alkaloid for probing microglial signaling and for guiding the rational development of structurally related derivatives with defined neuroimmune-modulatory properties.

In summary, arecoline, the major alkaloid of Areca catechu, demonstrated multi-target suppression of microglial inflammatory signaling in vitro. At non-cytotoxic concentrations, arecoline reduced NO and cytokine production and inhibited activation of both the TLR4/NF-κB and PI3K/AKT pathways, revealing a broader regulatory profile than previously recognized. These findings provide mechanistic insight into how a bioactive alkaloid shapes microglial activation and highlight the scientific significance of arecoline as a pharmacologically active scaffold for dissecting neuroimmune signaling. Rather than serving as a direct therapeutic candidate, arecoline holds value as a mechanistic probe and as a structural template for developing safer derivatives that may retain the beneficial anti-inflammatory signaling properties identified here. Several limitations should be acknowledged. BV2 microglia provide a widely used platform for mechanistic studies, yet reliance on a single immortalized cell line does not fully capture the complexity of primary or human microglia. Validation using primary microglial cultures or human microglial models will therefore be essential to determine the generalizability of these findings. Although GAPDH remained stable across experimental groups, previous reports indicate that GAPDH expression may be affected by oxidative or NO-related metabolic stress; future work will incorporate additional loading controls such as α-tubulin or Cyclophilin A to further strengthen quantitative rigor. Moreover, in vivo studies are required to assess pharmacodynamics, therapeutic windows, and systemic safety, and to evaluate structurally modified arecoline analogues with improved selectivity. Overall, these results expand the pharmacological relevance of plant-derived alkaloids in neuroinflammation research and position arecoline as a structurally informative and mechanistically valuable scaffold for further exploration in neuroimmune-related disorders.

## 4. Materials and Methods

### 4.1. Materials and Equipment

BV2 cells were obtained from Wuhan Shine Biotechnology (Wuhan, China). Arecoline (≥98%, CAS No. 63-75-2, MW 155.20 g/mol) was supplied by Yuanye Biotechnology (Shanghai, China) and dissolved in dimethyl sulfoxide (DMSO) to form a stock solution. Working concentrations were freshly prepared in DMEM, ensuring DMSO levels remain under 0.1% (*v*/*v*). LPS was obtained from Sigma-Aldrich (St. Louis, MO, USA).

CCK-8, NO assay kit, and BCA protein assay kit were purchased from Beyotime Biotechnology (Shanghai, China). ELISA kits for TNF-α, IL-1β, and IL-6 were supplied by Nanjing Jiancheng Bioengineering Institute (Nanjing, China). TRIzol reagent, cDNA synthesis kits, and TB Green^®^ Premix Ex Taq II were obtained from Takara Bio Inc. (Dalian, China).

Primary antibodies against GAPDH, COX-2, NF-κB p65, phospho-p65, PI3K p85, AKT, phospho-AKT, TLR4, and iNOS were purchased from Cell Signaling Technology (Danvers, MA, USA) or Proteintech (Wuhan, China). Secondary antibodies were obtained from Jackson ImmunoResearch (West Grove, PA, USA).

Primary laboratory equipment used in this study included CO_2_ incubator (Thermo Fisher Scientific, Waltham, MA, USA), refrigerated centrifuge (Zhongke Meiling, Hefei, China), autoclave sterilizer (GR60DR, Zhiwei, Shanghai, China), SpectraMax 190 microplate reader (Molecular Devices, San Jose, CA, USA), 2720 PCR thermal cycler (Applied Biosystems, Foster City, CA, USA), LightCycler 480 II real-time PCR system (Roche, Basel, Switzerland), Mupid electrophoresis equipment (Takara Bio, Shiga, Japan), BV-2 and BT-2 gel systems (Tanon, Shanghai, China), and an EUV imaging system (Korea Biotech, Seoul, Korea). Additional instruments included a DHG-9240A drying oven (Yiheng, Shanghai, China), a JY92-11N ultrasonic cell disruptor (Ningbo Xinzhi Biological, Ningbo, China), a TG-600 high-speed refrigerated centrifuge (Xiangyi, Changsha, China), and a V370 scanner (Epson, Nagano, Japan).

### 4.2. Culture Conditions and Experimental Procedures

BV2 microglial cells were grown in Dulbecco’s Modified Eagle Medium (DMEM) containing 10% fetal bovine serum, 100 U/mL penicillin, and 100 μg/mL streptomycin. These cultures were maintained at 37 °C in a humidified environment enriched with 5% CO_2_. Once the cells reached 70–80% confluence, they were split at a 1:3 ratio and utilized between the third and tenth passages.

For arecoline treatments, the compound was diluted in serum-free DMEM immediately before use. BV2 cells were seeded into culture plates and allowed to adhere overnight before being divided into experimental groups: (1) Control group (untreated), (2) LPS group (1 μg/mL), (3) Arecoline treatment groups (10, 20, and 40 μmol/L), and (4) Arecoline + LPS groups (Arecoline at 10, 20, or 40 μmol/L combined with 1 μg/mL LPS). Arecoline was applied 2 h prior to LPS stimulation based on established BV2 pre-treatment protocols that commonly use a 1–3 h window to ensure effective intracellular activity [70].

Preliminary CCK-8 assays confirmed that arecoline concentrations ≤40 μmol/L had no cytotoxicity, whereas ≥50 μmol/L significantly decreased cell viability. Consequently, 10–40 μmol/L were selected for subsequent experiments.

### 4.3. Determination of Cell Viability Using the CCK-8

The assessment of cell viability was carried out via the CCK-8 assay. BV2 cells were plated in 96-well dishes at a density of 5 × 10^4^ cells per milliliter, with 100 microliters allocated to each well, and left to settle overnight. After the experimental interventions, a 10 μL aliquot of CCK-8 solution was introduced into every well, and the plates were incubated in a cell incubator for 2 h. Measurements of absorbance were taken at 450 nm with a microplate reader.

### 4.4. Determination of NO Generation

NO levels were measured as a widely used quantitative indicator of LPS-induced microglial inflammatory activation [71]. Nitrite in culture supernatants was quantified using the Griess colorimetric reaction. After treatment, supernatants were centrifuged (1200 rpm, 3 min). 50 μL samples were combined with Griess reagents in 96-well plates. Subsequent measurement of absorbance at 540 nm facilitated the quantification of nitrite concentrations in the samples.

### 4.5. Quantification of Cytokines Using ELISA

Commercial ELISA kits quantified TNF-α, IL-6, and IL-1β levels in culture medium following the protocols provided by the manufacturers.

### 4.6. Determination of mRNA Expression by Quantitative Real-Time PCR

Total RNA was extracted from BV2 cells using TRIzol Reagent. To assess RNA concentration and purity, we turned to spectrophotometric analysis, while sample integrity received the green light following agarose gel electrophoresis. To rule out any genomic DNA contamination, we treated our RNA samples with DNase I at 37 °C for half an hour. Next, we converted 2 μg of the purified total RNA into first-strand complementary DNA using M-MLV reverse transcriptase and oligo(dT)_18_ primers, putting an end to the enzymatic activity by heating the mixture to 70 °C for 15 min.

The process of quantitative amplification was conducted on a Roche LightCycler^®^ 480 II machine with the TB Green^®^ Premix Ex Taq II kit. The reaction mixture comprised diluted cDNA template, primer pairs, a 2× qPCR premix, and nuclease-free water. The cycling procedure kicked off with a 5-min denaturation blast at 95 °C, before diving into 40 rounds of alternating 20-s denaturation at 95 °C and a 60-s combined annealing/extension phase at 60 °C. The primer sequences for the amplification are detailed in Table 1.

Primer efficiencies were validated by standard-curve analysis and ranged between 90% and 110%. Melt-curve analysis was performed for every reaction, and all primer pairs produced a single, sharp peak, confirming specific amplification without primer-dimer formation. Relative gene expression levels were calculated using the 2^−ΔΔCt^ method after normalization to β-actin.

### 4.7. Protein Expression Analysis by Western Blotting

Following treatment, BV2 cells were collected and given two washes with chilled phosphate-buffered saline before being disrupted in RIPA buffer enhanced with a comprehensive mix of protease and phosphatase inhibitors. The resulting lysates were kept on ice for half an hour, followed by centrifugation at 12,000 rpm for 15 min at 4 °C to isolate the supernatant. Total protein concentration was quantified using a BCA assay kit. Identical protein quantities (30 μg per sample) underwent separation via SDS-PAGE and were transferred to PVDF membranes featuring 0.45 μm pores. After a preliminary one-hour blocking stage with 5% bovine serum albumin at room temperature, the membranes were left to incubate overnight at 4 °C. During this period, they were exposed to specific primary antibodies targeting TLR4, COX-2, iNOS, NF-κB p65, phosphorylated p65, PI3K, AKT, phosphorylated AKT, and GAPDH. Post-irrigation with the TBST solution, membranes underwent one-hour incubation with conjugated secondary antibodies. Protein bands were visualized using an enhanced chemiluminescence kit and documented with a gel imaging system. Band intensity quantification was performed with Image-Pro Plus 6.0 (Rockville, MD, USA). For comprehensive details regarding the primary antibodies and their corresponding dilution percentages, refer to Table 2.

### 4.8. Statistical Analysis

Data are presented as mean ± SEM. Statistical analyses were performed using GraphPad Prism 9.0 (San Diego, CA, USA). Comparisons among groups were made by one-way analysis of variance (ANOVA) followed by Tukey’s multiple-comparison test. Two-tailed *p* values < 0.05 were considered statistically significant.

## 5. Conclusions

In conclusion, arecoline attenuates LPS-induced pro-inflammatory responses in BV2 microglia by reducing nitric oxide release and suppressing cytokine production. Mechanistically, these effects are linked to inhibition of TLR4/NF-κB and PI3K/AKT signaling, indicating that arecoline functions as a multi-target regulator of microglial immune signaling. The identification of this dual-pathway regulation expands current understanding of arecoline’s biological activity and provides a potential structural basis for designing blood–brain-barrier-permeable neuroprotective derivatives. Further in vivo and toxicological evaluations will be necessary to clarify its safety profile and therapeutic feasibility in neuroinflammation-related disorders.

## Figures and Tables

**Table 1:** Primer sequences for qPCR analysis in BV2 microglial.

Gene	Forward Primer (5′→3′)	Reverse Primer (5′→3′)
β-actin	GAGATTACTGCTCTGGCTCCTA	GGACTCATCGTACTCCTGCTTG
TNF-α	TAACTTAGAAAGGGGATTATGGCT	TGGAAAGGTCTGAAGGTAGGAA
IL-6	TTGCCTTCTTGGGACTGATG	ACTCTTTTCTCATTTCCACGATTT
IL-1β	TCACAAGCAGAGCACAAGCC	CATTAGAAACAGTCCAGCCCATAC

Primer sequences were designed according to Mus musculus gene sequences retrieved from the NCBI database.

**Table 2:** Primary antibodies used for Western blotting and their dilution ratios.

Antibody Target	Primary Antibody	Dilution Ratio	Molecular Size
1	PI3K	1:1000	85 kDa
2	p-AKT	1:1000	60 kDa
3	AKT	1:1000	60 kDa
4	p-p65	1:1000	65 kDa
5	p65	1:500	65 kDa
6	iNOS	1:500	131 kDa
7	COX2	1:1000	74 kDa
8	TLR4	1:1000	96 kDa
9	GAPDH	1:1000	37 kDa

All primary antibodies were rabbit-derived.

## Data Availability

The original contributions presented in this study are included in the article. Further inquiries can be directed to the corresponding authors.

## Abbreviations

The following abbreviations are used in this manuscript:

LPS	Lipopolysaccharide
NO	Nitric oxide
DMEM	Dulbecco’s Modified Eagle MediumDulbecco
DMSO	Dimethyl sulfoxide
BCA	Bicinchoninic acid
BSA	Bovine serum albumin
ELISA	Enzyme-linked immunosorbent assay
qPCR	Quantitative real-time polymerase chain reaction
IL-1β	Interleukin-1 beta
IL-6	Interleukin-6
TNF-α	Tumor necrosis factor-alpha
TLR4	Toll-like receptor 4
NF-κB p65	Nuclear factor kappa-light-chain-enhancer of activated B cells p65 subunit
p-p65	Phosphorylated NF-κB p65
PI3K p85	Phosphoinositide 3-kinase regulatory subunit p85
AKT	Protein kinase B
p-AKT	Phosphorylated AKT
iNOS	Inducible nitric oxide synthase
COX-2	Cyclooxygenase-2
GAPDH	Glyceraldehyde-3-phosphate dehydrogenase
